# Modulation of Epithelial to Mesenchymal Transition Signaling Pathways by *Olea Europaea* and Its Active Compounds

**DOI:** 10.3390/ijms20143492

**Published:** 2019-07-16

**Authors:** Rabiatul Adawiyah Razali, Yogeswaran Lokanathan, Muhammad Dain Yazid, Ayu Suraya Ansari, Aminuddin Bin Saim, Ruszymah Bt Hj Idrus

**Affiliations:** 1 Department of Physiology, Faculty of Medicine, Universiti Kebangsaan Malaysia Medical Centre, Universiti Kebangsaan Malaysia, Cheras, 56000 Kuala Lumpur, Malaysia; 2 Tissue Engineering Centre, Faculty of Medicine, Universiti Kebangsaan Malaysia Medical Centre, Universiti Kebangsaan Malaysia, Cheras, 56000 Kuala Lumpur, Malaysia; 3 Ear, Nose & Throat Consultant Clinic, Ampang Puteri Specialist Hospital, Ampang, 68000 Selangor, Malaysia

**Keywords:** olive, Mediterranean diet, EMT, fibrosis, natural product, signaling pathway

## Abstract

Epithelial-mesenchymal transition (EMT) is a significant dynamic process that causes changes in the phenotype of epithelial cells, changing them from their original phenotype to the mesenchymal cell phenotype. This event can be observed during wound healing process, fibrosis and cancer. EMT-related diseases are usually caused by inflammation that eventually leads to tissue remodeling in the damaged tissue. Prolonged inflammation causes long-term EMT activation that can lead to tissue fibrosis or cancer. Due to activation of EMT by its signaling pathway, therapeutic approaches that modulate that pathway should be explored. *Olea europaea* (OE) is well-known for its anti-inflammatory effects and abundant beneficial active compounds. These properties are presumed to modulate EMT events. This article reviews recent evidence of the effects of OE and its active compounds on EMT events and EMT-related diseases. Following evidence from the literature, it was shown that OE could modulate TGFβ/SMAD, AKT, ERK, and Wnt/β-catenin pathways in EMT due to a potent active compound that is present therein.

## 1. Introduction

Epithelial-mesenchymal transition (EMT) is a dynamic process involving polarized epithelial cells that lose their cell-cell adhesion while progressively undergoing phenotypic changes to become mesenchymal phenotypes [1]. EMT plays a critical role in various physiological events in the human body, and is necessary for proper re-epithelization and extracellular matrix deposition. However, prolonged EMT may results in fibrosis, cancer formation and invasion.

EMT can be classified into three different settings with distinct functional consequences [2,3]. Type 1 EMT occurs during organ formation and embryogenesis, where cells undergo EMT and mesenchymal-epithelial transition (MET) mutually to form a functioning tissue or organ. Type 2 EMT is associated with inflammation, wound healing, tissue repair and regeneration, which helps with tissue reconstruction after injury. However, continual transition of epithelial cells causes fibrosis. Type 3 EMT is connected to genetic and epigenetic events, which contribute to cancer progression and metastasis. Even though these classifications are distinct from one another, there are a common set of genetics, biochemical elements and signaling pathway that cause these EMT events [2]. 

The underlying molecular process of EMT events subsequently cause polarized immotile epithelial cells to change their phenotype to motile mesenchymal cells. In order to become motile, transition from polarized epithelial cells to mesenchymal cells starts with decreased expression of a protein that is involved in cell-cell contact, i.e., E-cadherin. Concurrently, the expression of mesenchymal markers such as vimentin, N-cadherin and fibronectin increases, which eventually leads to cell motility and invasion. Several studies have shown that there are various signaling pathways involved in EMT, such as TGF-β1, ligand binding through the RTK receptor (FGF, EGF, HGF), Wnt/beta-catenin and Notch signaling pathways [4,5,6]. These signaling pathways are important for EMT events to occur. 

Recently, several studies have reported the ability of natural products to modulate EMT signaling pathways [7,8]; this has been described extensively in a review by Zhang et al. [9]. Various types of natural products, such as grapes, as well as turmeric and its active compounds (resveratrol and curcumin), are able to regulate the signaling pathway of EMT, i.e., the MAPK/ERK and TGFβ1/SMAD pathways [9,10,11]. Therefore, targeting EMT signaling pathways to modulate prolonged EMT-related diseases such as fibrosis and inflammation might open new horizons in tackling these issues.

*Olea europaea*, or olives, belong to the Oleaceae family and are known to be a promising natural product with abundant health benefits due to the presence of phenolics and flavonoids [12,13]. The Mediterranean population, which is known to has the lowest incidence of chronic inflammatory disease in the world, consumes a Mediterranean diet that includes olives [14]. This has led to various research efforts on the benefits of olives to human health, through which it was demonstrated that olives have anti-inflammatory properties [15,16,17], can be nutraceutical agents [18], have anti-carcinogenic activity [6,19,20,21], help in reducing atherosclerosis [12,22], have a protective role against excessive fat accumulation [23], and are able to reduce cardiovascular risk [17]. Therefore, it can be hypothesized that olives are presumably able to modulate the EMT pathway. In this review, we explore the ability of *Olea Europaea* and its active compounds in modulating the EMT signaling pathway.

## 2. *Olea Europaea*

Archaeological evidence has revealed olive trees as one of the oldest known cultivated trees, dating back to 6000 BC [24]. Various scriptures, such as the Quran and the Bible, have mentioned olives as precious fruit; olive oil has also been considered sacred by some religions. Traditionally, olives have been used as traditional medicine for a wide range of ailments in various countries. In Morocco, olive fruit has been used to treat respiratory infections, stomach diseases and diarrhea [25]. Meanwhile, in Greece, the boiled extract of fresh leaves is taken orally to treat asthma and high blood pressure [26]. Overall, this points to the fact that olives have been well established for ages as a natural therapeutic product that helps to treat ailments, both directly or indirectly. Considering the beneficial evidence on human health, it seems that olives are a natural product that can potentially be used in modulating EMT-related diseases.

Olive fruit is a fleshy drupe with a thin skin and a hard pit. It is usually consumed as table olives or in the form of oil, but is rarely consumed in its raw form due to its bitterness. The bitterness comes from its phenolic compounds (oleuropein and ligstroside) which must be removed to make olive fruit palatable. Olive fruits are usually processed in order to make them less bitter, especially for table olives, by hydrolyzing oleuropein via chemical or enzyme catalysis [27]. In addition, olives have a high oil content, so the fruit is pressed to acquire olive oil. There are several types of olive oil including extra virgin olive oil (EVOO), virgin olive oil (VOO), ordinary virgin olive oil (OVOO), lampante virgin olive oil, refined olive oil, olive oil and olive-pomace oil [24] which are different from one another in terms of content, due to the method of procuring oil from the olive fruit.

The plant’s secondary metabolites consist of various compounds that are not associated with its growth; however, they have other important functions such as defense, and provide the plant with its characteristics, e.g., color. Phenolic compounds, a type of secondary metabolite, can be characterized by the presence of at least one hydroxylated aromatic ring. Besides having high amounts of mono-unsaturated and poly-unsaturated fatty acids, olive oil also contains beneficial phenolic compounds [28]. However, the phenolic content obtained from olive fruit varies from batch to batch due to several factors, such as the oil extraction method, climate, olive cultivation region, and degree of olive maturation [27,29]. There are at least 36 phenol compounds in olive oil [30] that can be categorized according to their chemical structures. Among known lipophilic phenols is tocopherols (vitamin E), while hydrophilic phenols include phenolic acids (phenol carboxylic acid), phenolic alcohols, flavonoid and secoiridoids [12,27,28]. 

Phenolic acids such as syringic, benzoic, vanillic and cinnamic acids, among others, have been identified in virgin olive oil as simple phenols that have a strong influence on oil flavor and that can also play a biochemical role as antioxidants [31]. Caffeic acid, chlorogenic acids (ferulic, vanillic, coumaric, and syringic) and the more complex caffeic acid sugar ester verbascoside are the phenolic acids that predominate in olive fruit.

The most common phenolic alcohols in olive fruit are hydroxytyrosol and tyrosol. Hydrolysis of oleuropein will generate hydroxytyrosol, whereas hydrolysis of ligstroside will give rise to tyrosol. The potential therapeutic effect of hydroxytyrosol has been covered extensively in a review by Hu et al. [22], where they reported that hydroxytyrosol has anticancer, cardioprotective and neuroprotective potential, besides being a natural antioxidant [22]. 

Flavonoids are reported to have antioxidant and anti-inflammatory activities and the ability to decrease cancer disease risk [27,32]. Apigenin-7-glucoside, cyanidin-3-glucoside, cyanidin-3-rutinoside, luteolin-7-glucoside, luteolin, quercetin-3-rhamnoside, and rutin are the predominant flavonoids in olives. 

The most common secoridoids in olives are dimethyl-oleuropein, oleuropein and ligstroside. The aforementioned secoridoids have related chemical structures where oleuropein is an ester consisting of hydroxytyrosol and elenolic acid; meanwhile, ligstroside is an ester consisting of tyrosol and elenolic acid; other derivatives which are worthy of mention include oleuropein aglycone, elenolic acid, oleoside-11-methyl ester, and the dialdehydic form of elenolic acid linked to tyrosol (3,4-DHEA- EDA) [27]. Oleocanthal, are also one of the notable secoridoids from olive oil, which is known for its anti-inflammatory, antioxidative, antimicrobial, anticancer and neuroprotective activities. The biological activities of oleocanthal from a molecular perspective have been extensively reviewed by Pang et al. [33], where the ability of oleocanthal to suppress breast, liver, and colon cancer cells has been discussed.

## 3. Epithelial-Mesenchymal Transition

### 3.1. Physiological EMT

Diseases and the invasion of a foreign body usually cause an inflammatory assault which will lead to tissue remodeling in response to repairing and healing the wound. This dynamic process will cause changes in the epithelial cell phenotype and environment. Increased matrix production, matrix degradation, and subepithelial base membrane thickening were observed during tissue remodeling. Other than that, dedifferentiation of epithelial cells occurs when the cells change their phenotype from epithelial to mesenchymal through a normal reversible EMT mechanism [34,35]. 

During the re-epithelization process in wound healing, epithelial cells at the border of the wound will undergo partial EMT, at which point they become motile. Epithelial cells that once have polarity and have tight junction with adjacent cells, will start to show a reduced expression of junctional proteins, such as E-cadherin, ZO-1, claudins and occludin. Eventually, the epithelial cells will start to separate from one another. Besides alteration in the epithelial and junction marker, these dedifferentiated epithelial cells acquire mesenchymal phenotypes, such as expression of vimentin and α-SMA, due to rearrangement of cytoskeletal filaments that will prepare them to migrate. Dedifferentiated epithelial cells become enlarged and migrate across the wound bed where epidermal growth factor stimulates them to divide and proliferate, forming a new epidermal layer [36,37]. The re-epithelization process is halted once the wound repair is completed.

### 3.2. Pathological EMT

Fibrosis occurs when there is overgrowth, hardening and scarring of tissues; it is characterized by an excessive deposition of extracellular matrix components such as collagen [38]. Current treatments for fibrotic diseases such as idiopathic pulmonary fibrosis target the inflammatory response; however, there are converging lines of evidence showing that the mechanisms that cause fibrogenesis are not limited to the inflammation response. There is evidence showing that the myofibroblast can be generated from various sources, including resident mesenchymal cells, as well as epithelial and endothelial cells that undergo EMT [38]. 

During wound repair, dedifferentiated epithelial cells can migrate down to the lamina reticularis or subepithelial basal membrane, where they become myofibroblasts that synthesize matrix and collagens, causing the basement membrane to thicken and the wound bed to constrict [37,39]. Accumulation of myofibroblasts that secrete an excessive amount of collagen fiber can compromise the organ function, which will lead to its failure. In normal wound healing, myofibroblasts will undergo apoptosis once re-epithelization is complete [40]. However, prolonged myofibroblast activity will cause alterations in the composition and organization of an organ, which will lead to fibrogenesis [35,37]. Fibrosis of multiple organs, including those of the respiratory system, is believed to be caused by the impairment in the regulation of injury-triggered EMT [37]. Besides fibrosis, EMT was also observed in cancer cells such as colon, liver and prostate cancers [41,42,43]. Cancer cells undergoing EMT may invade and metastasize, causing manifestations of cancer that can be life-threatening. 

Overall, EMT mechanisms have outcomes that are both beneficial and deleterious. Even though the outcome of EMT can be distinct, from physiological to pathological, there are a common set of pathways behind it, which enable these diversiform phenotypic changes. Various molecular events cause the initiation of EMT, including activation of transcription factors leading to specific cell surface proteins expression, which eventually cause reorganization and expression of cytoskeletal proteins, ECM-degrading enzyme production, and changes in the expression of specific microRNAs.

### 3.3. Transcription Factors Influencing EMT

The signaling pathways that control EMT are multifaceted. Changes in epithelial phenotype in cells undergoing EMT are initiated by master regulators, including SNAIL, TWIST and Zinc finger E-box-binding (ZEB) transcription factor. There are controlled by each other and further define EMT by repression of epithelial-associated genes and the activation of mesenchymal-associated genes [1,44]. For instance, SNAIL represses E-cadherin by activating TWIST expression, subsequently activating EMT.

SNAIL family transcription factors play a crucial role in EMT. SNAIL is regulated by the GSK-3β signaling pathway via a series of phosphorylation events. SNAIL1 act as the E-cadherin transcriptional repressor when it binds to the E-cadherin promoter region [45,46]. Therefore, the accumulation of SNAIL1 in the nucleus is associated with lower E-cadherin expression. E-cadherin expression is reversible due to the bivalent chromatin domain of the E-cadherin promoter, which directs both repression and activation of E-cadherin [1]. Besides, SNAIL also activates genes that contribute to the mesenchymal phenotype, such as N-cadherin [3].

TWIST belongs to the homodimeric and heterodimeric basic helix–loop–helix (bHLH) family of transcription factors; its expression downregulates epithelial gene expression and activates mesenchymal gene expression [1,3]. Other than that, this transcription factors also plays an important role in cancer metastasis by influencing the expression of miRNAs that cause effects on HOXD1 downstream target genes, such as Rhoc, which plays critical role in cytoskeletal reorganization and cancer metastasis [47].

**Figure 1:**
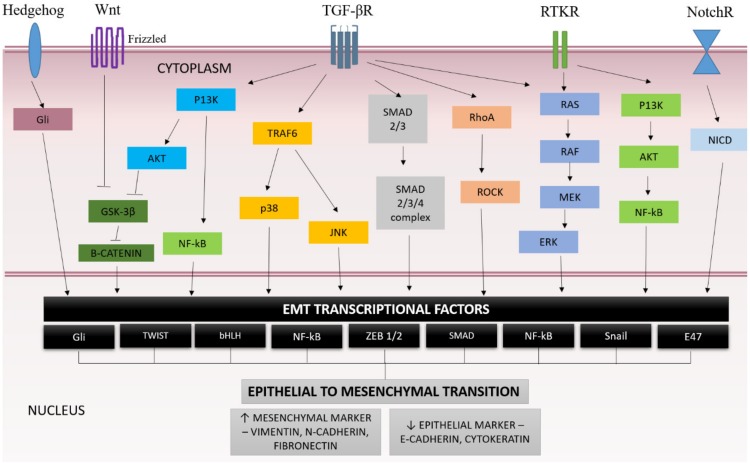
Signaling pathways regulating epithelial-mesenchymal transition (EMT) mechanisms [1,3,44].

Nevertheless, gene expression during EMT is regulated by various transcription factors which are expressed in response to activation of various signaling pathways by signal molecules, such as growth factors that bind through receptor, including the transforming growth factor family of receptors (TGFβ) and tyrosine kinase receptors (RTKs) [48,49]. Activated receptors subsequently activate intracellular signaling proteins that initiate a signaling cascade. These pathways might be activated at the same time or may crosstalk to each other, which leads to epithelial cell reprogramming (Figure 1).

### 3.4. EMT Signaling Pathway

The TGFβ signaling pathway is the most important and well-characterized pathway involved in EMT. In mammals, the TGFβ protein family consists of the TGFβ subfamily (TGFβ1, 2 and 3), activin and inhibin subfamilies, many bone morphogenetic proteins (BMPs) and other homodimers and heterodimers of ligands that bind to the transmembrane dual-specificity kinase receptor, which is the TGFβ receptor [1,50]. TGFβ1 regulates EMT in most cancer and fibrosis events. The pathways that involves TGFβ can be divided into two types: the SMAD-dependent and the SMAD-independent pathways.

#### 3.4.1. SMAD-Dependent Pathway 

Upon binding of the TGFβ ligand to its receptor, phosphorylation of SMAD2 and SMAD3 occurs, and the SMAD2/SMAD3 dimer will later form trimmers with SMAD4. After that, the complexes translocate into the nucleus and bind with DNA-binding transcription factors, such SNAIL, ZEB and TWIST [3]. This will induce the transcription of genes associated with EMT. SMAD complexes bind to SNAIL1 promoter to activate SNAIL1 transcription and facilitate SNAIL1 in repression of genes encoding E-cadherin and occludin [51]. The expression and activity of other transcription factors such as ZEB and TWIST also increased after being induced by TGFβ [1]. All of this causes the downregulation of epithelial markers (E-cadherin and cytokeratins) and the upregulation of mesenchymal markers (vimentin, N-cadherin, and fibronectin) [1]. Crosstalk of TGFβ with other signaling pathways like Notch, Wnt/β-catenin, and pathways that are activated by ligand binding to RTK receptor, can also induce EMT, which further helps to retain the mesenchymal phenotype.

#### 3.4.2. SMAD-Independents Pathway

Other than the TGFβ SMAD-dependant signaling pathway, EMT signaling also can be activated through: (1) the TGFβ SMAD-independent pathway; (2) other signaling pathways, such as Notch and Hedgehog signaling pathways; or (3) by growth factors that act through tyrosine kinase receptors (RTKs), such as hepatocyte growth factor (HGF) and fibroblast growth factor (FGF).

TGFβ activates TGFβ SMAD-independent pathway signaling through RHO-like GTPases, PI3K and MAPK pathway [1,44]. Rho and ROCK activation cause cytoskeletal changes and actin reorganization, which contribute to EMT activation. The epithelial cells then start to become motile, eventually taking on a mesenchymal phenotype. Besides that, TGFβ also activates the AKT pathway through PI3K activation, which leads to an increase in cell size, protein synthesis, motility, invasion and transition from an epithelial to mesenchymal phenotype [52]. Activation of AKT increases the level of SNAIL1 expression, hence causing E-cadherin repression, and increases invasive behavior and metastatic potential. Additionally, TGFβ proteins also activate ERK, p38 MAPK and JUN N-terminal kinase (JNK) MAPK pathways. Activation of ERK or p38 MAPK kinase activity increases TGFβ-induced EMT-related gene transcription, increasing the expression of MMP and N-cadherin [1].

Ligand binding to RTKs also causes the activation of several similar TGFβ pathways, such as the PI3K/AKT pathway and ERK/MAPK pathway. Signaling via either MAPK or PI3K along with TGF-β are necessary and sufficient to regulate EMT [9]. The MAPK pathway is activated by Ras, and the MAPK family consists of ERK 1/2, ERK 5 and p38 protein. After binding with a ligand, RTK changes its conformation, phosphorylate tyrosine, and initiates Ras downstream pathway causing the activation of SNAIL1 and SNAIL2, thereby promoting cell motility in EMT [1,9]. 

There are other extracellular signaling types that regulate EMT such as Wnt, Hedgehog (Hh) and Notch signaling pathway. Wnt signaling is important for the regulation of EMT. In the absence of Wnt signaling, β-catenin will form complex with Axin, adenomatous polyposis coli (APC), glycogen synthase kinase-3β (GSK-3β), and casein kinase 1 (Ck1) in the cytoplasm [44]. However, upon activation of Wnt signaling, β-catenin stability is affected, causing its accumulation in the cytoplasm and enabling it to regulate gene expression. Subsequently, β-catenins are translocated to the nucleus and bind with transcription factor TCF/LEF, which increases the expression of SNAIL and ZEB, leading to activation of EMT by epithelial phenotype suppression [44]. Another pathway involved in EMT is the Hedgehog (Hh) pathway. The Hh family consists of three Hh proteins, which are Sonic Hedgehog, Desert Hedgehog and Indian Hedgehog. When Hh ligands bind to the Hh receptor, it causes upregulation of β-catenin and BMP, which will cause EMT activation. In addition, molecules involved in Wnt signaling, such as GSK-3β, also regulate Hh signaling, suggesting crosstalk between the two potential pathways [44]. Other than that, the presence of GLI protein is also one of the indications of Hh pathway activation [1,44]. Activated protein kinase A (PKA) binds to other kinases, such as GSK-3β, to phosphorylate GLIs in the cytosol. GLI proteins can promote the expression of EMT genes by physically binding to their promoting region, such as SNAIL1 [53], subsequently activating the EMT mechanism. Additionally, Notch signaling also plays a role in EMT; however, it requires coordination with other signals. TGFβ increases Notch activity through SMAD3, subsequently promoting SNAIL2 expression, which in turn suppresses E-cadherin. Cross-talk between Wnt and Notch pathways have also been observed to cause a tumorigenic phenotype [3].

The aforementioned signaling pathways have been demonstrated to be able to activate EMT mechanisms in cells, whether alone or by crosstalk with other pathways. Due to the fact that EMT is involved in inflammation and cancer, targeting the pathway might be one therapeutic approach that could influence the outcome of chronic inflammation diseases, such as fibrosis and rhinosinusitis.

## 4. Involvement of *Olea Europaea* and Its Active Compounds in Targeting Specific Signaling Pathway

It has been established that natural products, such as honey [54], curcumin [55], *Olea europaea* [56] and *Paeonia lactiflora* [51], have the ability to regulate EMT to prevent diseases, such as pulmonary fibrosis, liver fibrosis, renal fibrosis and cancer. These beneficial plants with active compounds act by targeting a specific molecular pathway in diseases. However, further study needs to be performed to explore the action of these natural products on the specific pathways causing EMT.

Numerous transcription factors are involved in the activation of EMT which may act synergistically using the same set of pathways. This activation will eventually lead to a decrease in epithelial-associated protein expression and an increase in mesenchymal-related protein expression. Various studies observed these transitions to prove that the tested natural product was able to regulate EMT. For example, a recent study performed by Wang et al. showed that oleanolic acid from olives inhibits the expression of mesenchymal-related protein (vimentin) while retaining the E-cadherin expression of hepatocellular carcinoma cell [57]. Other research on the action of *Olea europaea* and its active compounds on EMT pathway also reported the same pattern (Table 1); the affected pathway can be divided into two types: the SMAD-dependant and -independent pathways.

### 4.1. SMAD-Dependant Pathway

TGFβ1 has been known to be the major inducer of EMT. A study performed by Vazquez-Martin et al. [60] showed that the presence of phenolic secoiridoid in extra virgin olive oil (EVOO) caused phenotypic changes from fibroblastic to cobblestone in TGFβ1-treated kidney cells. Moreover, EVOO-derived crude phenolic extract also prevented disintegration of E-cadherin of TGFβ1-treated kidney cells at cell–cell contacts, prevented vimentin activation and decreased nuclear accumulation of SNAI2 in response to TGFβ1 [60]. The same study also investigated the ability of EVOO phenolics to impede breast cancer oncogenic EMT events. Besides promoting a more cuboidal appearance in a TGFβ1-treated breast cancer epithelial cell line, EVOO-derived crude phenolic extract significantly prevented TGFβ1 from upregulating SMAD4 and SNAIL2, hence allowing the cells to retain E-cadherin expression [60]. 

Another study by Lupinacci et al. [62], investigated the potential of olive leaf extract (OLE) in treating peritoneal fibrosis. This extract was revealed to have phenolic compounds similar to olive fruit, i.e., oleuropein, hydroxytyrosol and verbascoside, and were able to inhibit TGFβ1-induced EMT Met5A cell migration. Treatment of TGFβ1-induced EMT in Met5A cells with OLE decreased the fibrogenic expression markers (α-SMA, N-cadherin, vimentin and fibronectin) and reduced MMP2/MMP9 expression which are responsible for basement membrane degradation. The reduction of fibrogenic expression leads to reduction in migration and motility. The phosphorylated content of SMAD3 and SMAD4 in the nucleus and cytosol was also studied, showing the co-treatment of OLE + TGFβ1 reduced activation of phosphorylated SMAD3 and SMAD4 in both the cytosol and nuclear fractions of the cells. Besides that, there was a reduction in SNAIL expression levels, one of the most important transcriptional repressors of E-cadherin protein, leading to an increase in E-cadherin expression and its promoter activity in the OLE-treated group [62]. 

Additionally, the phenolic compounds of olives, oleanolic acid (OA), was found to decrease the phosphorylation of Smad2/3 and SNAIL in a dose-dependent manner in TGFβ1-treated kidney cells, suggesting that OA attenuates TGFβ1-induced EMT in kidney cells in association with the modulation of the TGFβ1/SMADS pathway [61].

The above findings suggest that olives and their active compounds could modulate the TGFβ1/SMAD signaling pathway and the expression of transcription factors (SNAIL) involved in E-cadherin promoter transactivation.

### 4.2. SMAD-Independent Pathway

#### 4.2.1. AKT 

EMT events are also involved in the metastasis of cancer cells, especially through AKT and MAPK/ERK signaling pathways. Oleuropein [63,65,69], oleocanthal [21,66,67,68,71] and hydroxytyrosol [72] were observed to cause downregulation of p-AKT in prostate cell lines, glioma cell lines, hepatic steatosis models, liver and colon cancer, non-melanoma skin cancer, myeloma-derived cell lines and human malignant melanoma cells. AKT is a downstream effector of P13K, and its activation promotes cell survival, enhanced cell invasion and cell cycle progression, especially in tumour cells [75]. 

An in vivo study of colorectal cancer (CRC) showed that oleuropein ameliorated clinical symptoms such as diarrhoea and rectal bleeding in CRC-induced mice. Oleuropein also decreased cell proliferation and reduced phosphorylated-AKT (pAKT) in the cytosol of CRC-induced mice. In addition, oleuropein also downregulated the proinflammatory biomarkers that are associated with wnt/β-catenin signaling pathway [63,74]. Based on the results of these studies, it was deduced that oleuropein has chemopreventive effect and might be able to modulate the AKT, ERK and wnt/β-catenin signaling pathway of EMT.

In glioma cell line, treatment of oleuropein caused apoptosis and decreased phosphorylation level of AKT, however it did not change the expression levels of phosphorylated p38, ERK and JNK. This finding suggested that oleuropein may induce pro apoptotic and anti-invasive effects through AKT signaling [63]. Other than that, the effect of oleuropein on a prostate cancer cell line was similar to the glioma cell line where oleuropein decreased prostate cancer cell proliferation and induced necrotic cell death. Treatment with oleuropein on a prostate cancer cell line caused a reduction of pAkt(Ser473) and Akt(Thr308), suggesting that oleuropein has an inhibitory effect on AKT signaling [65]. Despite both studies showing the ability of oleuropein to inhibit the AKT signaling pathway, in FFA-induced cellular steatosis cell, there were no effects on the expression of AKT, even though oleuropein reduced the FFA-induced cellular steatosis [69]. 

The activation of HGF/cMET signaling in mammary cancer cells caused the activation of downstream effector, AKT. Oleochantal has been shown to be able to block the activation of mammary cancer cells, and hence, to block PI3K and subsequent Akt-NFkB activation. Other than that, oleocanthal treatment on mammary cancer cells and human hepatocellular carcinoma suppressed mesenchymal marker, vimentin, and N-cadherin and stabilized ZO-1 and E-cadherin expression suggested that oleocanthal was able to modulate EMT mechanisms [21,73]. Oleocanthal and oleacein have also been shown to significantly reduce AKT phosphorylation compared to hydroxytyrol and tyrosol in non-melanoma skin cancer cell [66]. In malignant melanoma, besides causing a reduction in AKT phosphorylation, oleocanthal selectively inhibited cell growth of melanoma cell line, but not on human dermal fibroblast viability [67]. 

#### 4.2.2. MAPK/ERK 

Ligand binding to the RTK receptor leads to the activation of MAPK/ERK, which plays a role in EMT activation. However, treatment with olive or its active compounds seems to be able to modulate this RAS-RAF-MEK-ERK pathway. Oleocanthal, oleacein and hydroxytyrosol were observed to exhibit an inhibitory effect on ERK1/2 phosphorylation in a TGFβ1-treated mesothelial cell line, non-melanoma skin cancer cell line, myeloma-derived cell line and human melanoma cell line [62,66,67,68,69,70]. 

In a study by Lupinacci et al., besides showing that OLE has an effect on the SMAD-dependent pathway, they also studied the effect of OLE on SMAD-independent pathways of TGFβ1-induced EMT in Met5A cells. OLE cause reduced expression of ERK, JNK and p38 phosphorylation [62], which suggests that OLE has the ability to modulate both the SMAD-dependent and -independent pathways of EMT in peritoneal fibrosis cell.

Other than that, oleonalic acid also inhibited the EMT of glioma cells. The elevation of E-cadherin expression was observed, together with decreased expression of N-cadherin, Vimentin and Twist1. MAPK/ERK activation is related with EMT activation and progression in glioma cells. Furthermore, modulating EMT markers, oleonalic acid also suppressed the expression of MAPK/ERK signaling [64].

Polini et al. [66] observed that between four active compounds in olives (oleocanthal, oleacein, hydroxytyrosol and tyrosol), oleocanthal and oleacein have the greatest inhibitory activities on target signaling molecules, particularly on the B-Raf/ERK pathway in skin cancer cell. 

#### 4.2.3. Wnt/β-Catenin

Several studies have investigated the effect of olive and its active compounds on Wnt/β-catenin pathway. An in vivo study by Giner et al. showed that mice treated with oleuropein exhibited significantly lower levels of nuclear β-catenin in colon samples. It was also observed that oleuropein seems to have a blocking effect on the translocation of the key component of the Wnt pathway, β-catenin, along with a possible final modifying effect on β-catenin-related gene expression, as shown by the downregulation of COX-2 in colon tissue [74]. Hydroxytyrosol also demonstrated the ability to inhibit the Wnt pathway through an antagonist of the Wnt pathway, Sfrp4, that binds to the Frizzled receptors (Fzd) or to Wnt ligands, hence blocking the non-canonical and canonical β-catenin pathways [59].

**Figure 2:**
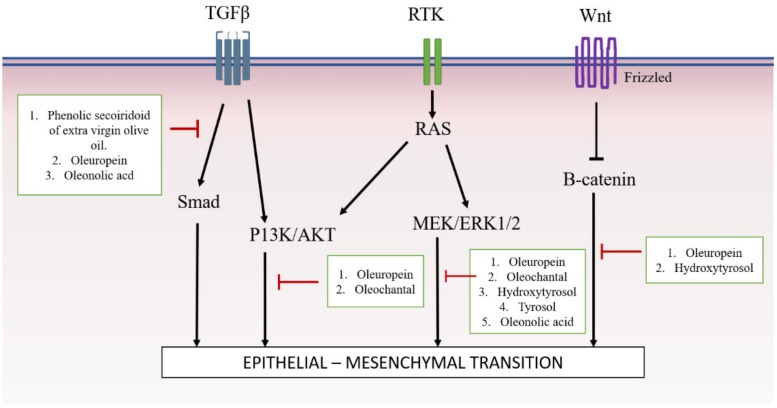
Summary of *Olea europaea* extract and its active compounds’ mechanism of action on signaling pathway of EMT.

Considering all of this evidence, it was shown that, due to potent active compounds present in *Olea europaea*, it is able to modulate the TGFβ/SMAD, AKT, ERK and Wnt/β-catenin pathways, and thus, inhibit the EMT mechanism (Figure 2).

## 5. Future Direction and Conclusions

*Olea europaea,* is a fruit with various phenolic compounds such as oleuropein and hydroxytyrosol. It has abundant health benefits, for example, anti-cancer and anti-inflammatory effects. Due to these properties, it is believed that *Olea europaea* could modulate EMT pathways, as EMTs are usually present in inflammation events and diseases like cancer and fibrosis.

Based on the evidence reviewed in this paper, it was demonstrated that olive and its active compounds can modulate EMT through several pathways. However, more studies need to be performed to further investigated this phenomenon. EMTs are classified into three different types but share a common set of pathways that regulate the EMT mechanisms. To date, no research has been done to differentiate the molecular events which occur in between these classifications. Other than that, most study on the effects of olives on EMT focus on cancer. There is limited research studying the effect of olives on other EMT-related diseases, such as fibrosis. Besides that, most associated research only used compounds such as oleuropein, oleochantal, hydroxytyrosol, and tyrosol. The effect of other types of olive-derived compounds should be explored to rigorously determine the effects of olives on EMT-related. Therefore, further research in various settings of EMT mechanisms should be explored and in vivo studies should be performed in order to move towards validating olives as a beneficial health supplement for human.

In this review we introduced the anti-EMT effects of *Olea europaea* and its active compounds that were shown to inhibit the MAPK/ERK1/2, PI3K/AKT, WNT/β-catenin and TGF-β1 pathways. As a way forward, further investigations should be undertaken on the mechanisms of action of *Olea europaea* and its active compounds in other EMT pathways and how they target EMT as a novel and global therapeutic approach to treat EMT-related diseases.

## Figures and Tables

**Table 1:** Summary of olive and its active compounds and target signaling pathway.

Type of Study	Disease	Pathways Involved	Subject/Cell Type/EMT Induction	Olive Product or Active Compound Concentration	Key Findings	References
In vitro	Breast cancer	EMT transcription factor	MCF-7	Oleuropein (OP) [50–1200 μg/mL]	Downregulated MMP-2, MMP-9, and ZEB1 Upregulated E-cadherin	[58]
		AKT MAPK	MDA-MB-231, MCF-7 and BT-474	(-)-Oleocanthal (OC) [0–40µM]	Increased E-cadherin and ZO-1 Decreased vimentin. Reduction of β-catenin expression.	[21]
		Wnt	BT549	Hydroxytyrosol [10, 25, 50, 75, and 100 μM]	Reduce Wnt co-receptor LRP6, Decrease β-catenin, SNAIL and SLUG and E-cadherin	[59]
	Breast cancer and renal fibrosis	SMAD	TGFβ1 treated-MCF-7 and MDCK	Extra virgin olive oil (EVOO) [200 ng/mL]	Prevented upregulations of SMAD4, SNAIL2, TCF4, VIM, FN and SERPINE1 genes.	[60]
	Renal interstitial fibrosis	SMAD	Renal proximal tubular epithelial cell line (NRK-52E)	Oleanolic acid (OA) [2, 4 and 8 μM]	Reversed all EMT markers Decreased the phosphorylation of Smad2/3, ILK and Snail in cells, which was initiated by TGF-β1	[61]
	Peritoneal fibrosis	SMAD ERK JNK p38 MAPK	TGFβ1-treated MeT-5A	Olive leaf extract (OLE) [high content of oleuropein] [25µg/mL]	Downregulated N-cadherin, α-SMA, fibronectin and vimentin. Inhibited cell migration. Decreased ERK, JNK and p38 phosphorylation	[62]
	Malignant glioma	A KT	Human malignant glioma cell line (U251 and A172)	Oleuropein (OP) [0, 200 and 400 μM]	Inhibit viability, supresses migration and invasion of glioma cells. Downregulated pAKT, No changes in expression levels of pp38, pERK and pJNK.	[63]
		MAPK/ERK	Human glioblastoma cell lines (U-87 MG, U-251 MG cells), primary glioma cells	Oleanolic acid (OA) [5, 10 and 25 mg/mL (10, 20 and 50 mM)]	Inhibited EMT Decreased N-cadherin, vimentin and TWIST1 Suppressed the activation of MAPK/ERK pathway	[64]
	Prostate cancer	AKT	Prostate cell lines (BPH-1, LNCaP, DU145)	Oleuropein (OP) [100–500 μM]	Downregulated pAKT, suggesting an inhibitory effect on AKT signaling	[65]
	Skin cancer (non-melanoma)	AKT ERK	Human epidermoid carcinoma cell line (A431) and human immortalized keratinocytes (HaCat)	Oleocanthal, oleacein, tyrosol and hydroxytyrosol [1–100 μM]	Decreased expression levels of B-Raf, pAKT and pERK proteins after treatment	[66]
	Skin cancer (malignant melanoma)	AKT ERK	Human melanoma cell line (A375)	Oleocanthal (OC) [0.01–50 µM]	Significant inhibition of ERK1/2 and AKT phosphorylation	[67]
	Multiple myeloma	AKT ERK	Myeloma-derived cell line (ARH-77, MOPC-31C)	Oleocanthal (OC) [25 and 50 µM]	Inhibitory activity on AKT and ERK1/2 phosphorylation Induced a phosphorylation of p38	[68]
	Non-alcoholic fatty liver disease (NAFLD)	AKT ERK	Free fatty acids (FFAs) induced HepG2 and FL83B	Oleuropein (OP) [10- and 50-μM]	Phosphorylation of ERK No phosphorylation of JNK or AKT.	[69]
	Colon cancer	ERK	Human colon adenocarcinoma cells (Caco-2)	Hydroxytyrosol [5.0–162.5 µM]	Strong inhibition of extracellular signal-regulated kinase (ERK)1/2 phosphorylation	[70]
	Colon and liver cancer	AKT ERK	Liver and colon cancer cells (HepG2 and Caco-2)	*Olea europaea* L. fruit extracts [0–1400 mg/mL]	Significantly decreased the pAKT in both cell lines pERK and p-p53 were markedly upregulated in both cell lines.	[71]
In vitro and In vivo	Hepatocellular carcinoma cell (HCC)	AKT	HCC cell lines HepG2, Hep3B and SK-HEP-1	Hydroxytyrosol [0–400 µM]	pAKT decreased	[72]
		JAK/STAT3	Huh-7, HepG2 and HCCLM3	(-)-Oleocanthal (OC) [0–80 μM]	Increased E-cadherin expression decreased N-cadherin, vimentin, Twist and pSTAT3 expression.	[73]
In vivo	Colorectal cancer	Wnt/β-catenin P13K/AKT	AOM/DSS-induced C57BL/6 mice CRC model	Oleuropein (OP) [50 or 100 mg/kg]	Exhibited lower levels of nuclear β-catenin AKT phosphorylation.	[74]
